# NanoTIO_2_ (UV-Titan) does not induce ESTR mutations in the germline of prenatally exposed female mice

**DOI:** 10.1186/1743-8977-9-19

**Published:** 2012-06-01

**Authors:** Anne Mette Zenner Boisen, Thomas Shipley, Petra Jackson, Karin Sørig Hougaard, Håkan Wallin, Carole L Yauk, Ulla Vogel

**Affiliations:** 1 The National Research Centre for the Working Environment, Copenhagen, Denmark; 2 National Food Institute, Technical University of Denmark, Søborg, Denmark; 3 Environmental Health Science and Research Bureau, Health Canada, Ottawa, Canada; 4 Department of Micro- and Nanotechnology, Technical University of Denmark, Lyngby, Denmark

**Keywords:** ESTR, Nanoparticles, Oogenesis, *In utero*

## Abstract

**Background:**

Particulate air pollution has been linked to an increased risk of cardiovascular disease and cancer. Animal studies have shown that inhalation of air particulates induces mutations in the male germline. Expanded simple tandem repeat (ESTR) *loci* in mice are sensitive markers of mutagenic effects on male germ cells resulting from environmental exposures; however, female germ cells have received little attention. Oocytes may be vulnerable during stages of active cell division (e.g., during fetal development). Accordingly, an increase in germline ESTR mutations in female mice prenatally exposed to radiation has previously been reported. Here we investigate the effects of nanoparticles on the female germline. Since pulmonary exposure to nanosized titanium dioxide (nanoTiO_2_) produces a long-lasting inflammatory response in mice, it was chosen for the present study.

**Findings:**

Pregnant C57BL/6 mice were exposed by whole-body inhalation to the nanoTiO_2_ UV-Titan L181 (~42.4 mg UV-Titan/m^3^) or filtered clean air on gestation days (GD) 8–18. Female C57BL/6 F1 offspring were raised to maturity and mated with unexposed CBA males. The F2 descendents were collected and ESTR germline mutation rates in this generation were estimated from full pedigrees (mother, father, offspring) of F1 female mice (192 UV-Titan-exposed F2 offspring and 164 F2 controls). ESTR mutation rates of 0.029 (maternal allele) and 0.047 (paternal allele) in UV-Titan-exposed F2 offspring were not statistically different from those of F2 controls: 0.037 (maternal allele) and 0.061 (paternal allele).

**Conclusions:**

We found no evidence for increased ESTR mutation rates in F1 females exposed *in utero* to UV-Titan nanoparticles from GD8-18 relative to control females.

## Background

Mutations in male and female gametes may lead to detrimental inherited effects in subsequent generations. Human exposure to particulate air pollution (PAP) has been shown to adversely affect germ cells in males [1]. Moreover, animal studies have demonstrated that inhalation of PAP can induce mutations in the male germline [2-6]. Airborne particles in the nanometer range deposit deep in the airways. These particles are cleared very slowly and a small fraction may translocate into the bloodstream [7,8]. Inhaled nanoparticles (NPs) are potent inducers of pulmonary inflammation and oxidative stress, which may affect the fetus indirectly during maternal exposure [9-11].

As a model of NP exposure we tested nanosized titanium dioxide (nanoTiO_2_) UV-Titan, which is used in the production of paints [9,12,13]. Large quantities of nanoTiO_2_ are used globally in a wide range of products. TiO_2_ was previously believed to be inert, but inhaled TiO_2_ has now been classified as possibly carcinogenic to humans by the International Agency for Research on Cancer [14]. TiO_2_ toxicity depends on particle size, crystalline form and surface modifications [15]. Pulmonary exposure to nanoTiO_2_ causes inflammation in rodents [9,16] and we recently found that a single UV-Titan instillation induced an inflammatory response in mice after 1 day [12,17]. In addition, UV-Titan particles remained in lungs 4 weeks after inhalation, causing long-lasting inflammation [9] .

Expanded simple tandem repeat (ESTR) *loci* in mice exhibit high spontaneous mutation rates enabling the study of induced germline mutations following environmental exposures. Radiation, air particulates, and a number of chemicals have been shown to increase ESTR mutations in male germ cells [2,5,18,19]. Very limited data exist on induced mutations in female germ cells, which have previously been considered highly resistant to genotoxicity [20]. However, oocytes could be vulnerable during stages of active cell division, i.e. during fetal development [20,21]. A recent study showed that prenatal exposure to 1 Gy of acute irradiation on GD12 resulted in a 1.94-fold increase in ESTR mutations in the offspring of irradiated female mice [22,23].

We hypothesized that prenatal exposure to NPs will affect female germline ESTR mutation frequency during stages of active cell division, similar to what has been found for male germline cells [1]. The present study investigates TiO_2_ nanoparticle-induced effects on female germline DNA by exposing pregnant female mice (P) to nanoTiO_2_ or clean filtered air *via* inhalation and subsequently mating their offspring (F1) with unexposed males. The observed F1 female germline ESTR mutation frequency was calculated by comparing allele size in the F2 offspring to their mother’s allele size to quantify repeat gains and losses.

## Methods

### Animals and exposure

**Figure 1:**
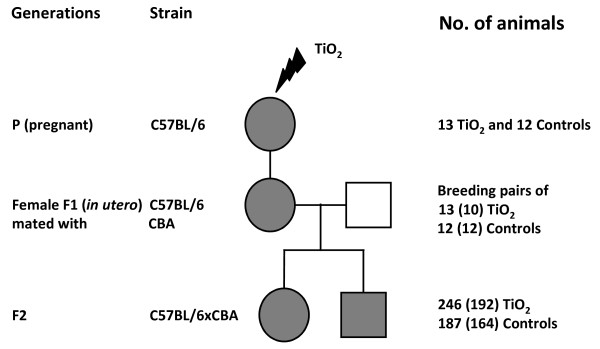
**Overview of the pedigree study.** Circles and squares represent female and male mice respectively. Grey symbols represent exposed animals and their descendants. White squares represent non-exposed CBA mates. Generation P pregnant mothers were exposed: 13 TiO_2_ exposed and 12 Controls. 246 F2 offspring were collected from TiO_2_ and 187 from Controls (number of successfully analyzed offspring 192 and 164, respectively).

All mice (Figure 1) were housed under controlled environmental conditions [9]. Generation P consisted of time-mated, nulliparous mice (C57BL/6JBomTac) exposed by whole-body inhalation to UV-Titan L181 (Kemira, Pori, Finland), a rutile TiO_2_ (70.8 wt.%) modified with 1.17 wt% zirconium, 12.01 wt% silicon, 0.60 wt% sodium oxide and 4.58 wt% aluminium. UV-Titanium is coated with polyalcohol adding to the remaining wt%. Primary particle size was 20.6 nm and surface area (BET) 107.7 m^2^/g. The particle number concentration in the exposure atmosphere was 1.70 ± 0.20·10^6^/cm^3^. The major particle size-mode was ~100 nm (geometric mean number diameter 97 nm). The mass-size distribution was strongly dominated by μm-size particles (geometric mean 3.2 μm) and 75% of the mass were represented by particles larger than 1.6 μm [9]. A detailed description of the physico-chemical characteristics of particle preparation, sample analysis and exposure monitoring of UV-Titan is reported in [9]. Mice were exposed to ~42.4 mg UV-Titan/m^3^ or filtered clean air on GD8-18, one h/day as described [9]. Generation P gave birth to generation F1 (C57BL/6JBomTac). At 19 weeks of age, 26 prenatally exposed F1 females (13 controls and 12 TiO_2_-exposed) were mated with unexposed CBA/J (Charles River, Sulzfeld, Germany) to produce generation F2 (C57BL/6 x CBA/J). A total of 450 F2 offspring (Figure 1) were collected for the present study. Mutation analysis and scoring were successful for 388 offspring. Procedures complied with EC Directive 86/609/EEC and Danish regulations on experiments with animals (Permission 2006/561-1123).

### DNA extraction and mutation analysis

F1 parents were euthanized after breeding, F2 offspring on postnatal day (PND) 2–7 or at maturity (PND80). F1 and F2 tail tissue was flash frozen in cryotubes (NUNC) in liquid N_2_ and stored at −80 °C. DNA was extracted by phenol-chloroform extraction and ESTR analysis was performed as in [2]. Briefly, 25 μg of mouse tail DNA was digested with *AluI* (New England BioLabs, Pickering, Ont.) at 37^o^ C overnight. F1 and F2 DNA samples were run on 40 cm long 0.8% agarose gels (SeaKem LE) for 48 hours in a cooled chamber at 130 V along with a 1 Kb ladder (Invitrogen, Burlington, Ont.). DNA was transferred to a nylon membrane by vacuum blotting (GE Osmonics, Minnetonka, MN) and hybridized to ^32^P-labeled *Ms6-hm* and *Hm2* probes [2]. F2 bands showing a shift of at least 1 mm relative to the F1 progenitor allele were scored as mutants. Bands were scored independently by 3 observers blinded to exposure status. Mutation rates were determined as the number of mutant bands per total number of bands scored (Table 1) and compared using a one-tailed Fisher’s exact test.

**Table 1:** Summary of ESTR mutation rates in F2 offspring of prenatally exposed female C57BL/6 mice

Group	**probe**	**N (F2 offspring)**	**Mutant bands**		**Mutation rate ± SEM (P value**^a^**)**	
			**Paternal**	**Maternal**	**Paternal**	**Maternal**
			**origin**	**origin**	**origin**	**origin**
Female controls	*Ms6-hm*	164	11	5	0.0671 ± 0.0002	0.0305 ± 0.0002
Female controls	*Hm-2*	164	9	7	0.0549 ± 0.0004	0.0427 ± 0.0004
Female controls	**Total**	**164**	**20**	**12**	**0.0610 ± 0.0028**	**0.0366 ± 0.0030**
Female TiO_2_ exposed	*Ms6-hm*	192	10	4	0.0521 ± 0.0004	0.0208 ± 0.0002
Female TiO_2_ exposed	*Hm-2*	192	8	7	0.0417 ± 0.0004	0.0365 ± 0.0003
**Female TiO**_**2**_**exposed**	**Total**	**192**	**18**	**11**	**0.0469 ± 0.0107 (P = 0.84)**	**0.0286 ± 0.0133 (P = 0.79)**

^a^ Fisher’s exact test 1-tailed.

## Results and discussion

F1 females were prenatally exposed to UV-Titan by maternal inhalation of 42.4 mg UV-Titan/m^3^ 1 hour/day on GD8-18 (Figure 1). 164 and 192 offspring from control and exposed females, respectively, were scored. Thus, a total of 328 and 384 inherited bands were scored per group. The observed mutation rate in germ cells of UV Titan-exposed F1 females was not significantly different from controls (Table 1). The *Ms6-hm* and *Hm-2* mutation rates in control females were similar to those found for females in other studies using the same mouse strain [2,19]. Furthermore, the number of offspring, sex-ratio and time to birth of the first F2 litter did not differ between groups, suggesting that UV-Titan did not affect viability of the F2 offspring (data not shown). Absence of effect is therefore not due to lower viability of affected offspring. Mutations in ESTRs should not affect offspring fitness since these *loci* do not have known functions.

ESTR mutations have been suggested to be induced *via* polymerase pausing resulting from the presence of epigenetic changes or DNA damage such as oxidative stress, strand breaks or adducts elsewhere in the genome rather than by direct DNA damage [5]. We have reported that the inhalation of a total dose of 840 μg UV-Titan per animal at GD8-18 induced persistent inflammation in the lungs of the time-mated P generation (Figure 1) [9,24]. Furthermore, 476 genes were found to be differentially expressed in the liver of newborn F1 generation females prenatally exposed to UV-Titan. We hypothesize that the transfer of inflammatory cytokines across the placenta may have caused this differential gene expression [10] since no TiO_2_ was detected in maternal liver, mother’s milk or offspring liver [9].

ESTR mutation analysis is a sensitive method, enabling analysis under realistic exposure scenarios. An *a priori* power analysis showed that group size in the present study provided a 77% chance of detecting a 2-fold increase in ESTR mutations at the 5% significance level. The exposure and the estimated inhaled dose of 840 μg used in this study is comparable to the permissible exposure limit by Danish Regulation and the exposure route (inhalation) is also relevant to environmental exposure [9]. As little as 54 μg UV-Titan can induce inflammation in mouse lungs after one day [12,17]. Female germ cells enter meiotic prophase on ~ GD13.5 [21]. In the present study female mice were prenatally exposed from GD8-18 ensuring that the period of mitotic germ cell division was targeted; these mothers were exposed to ~458 μg prior to GD13. Consequently, a high degree of inflammation was likely to be present at GD13.5, when oocytes cease to be susceptible to ESTR mutations [21,22].

In parallel with the present study (in the same laboratory and time period), ESTR germline mutations in male and female mice prenatally exposed to diesel exhaust particles (DEP) by inhalation were quantified [2]. Male germ cell mutation rates were significantly increased following exposure to DEP and may thus be regarded as a positive control for the ability to detect induced ESTR mutation. ESTR mutation rates were not significantly increased in germ cells of females prenatally exposed to DEP. To our knowledge, this is the only other study, which has investigated chemically induced ESTR mutations in prenatally exposed females. A recent study showed that dividing oocytes are susceptible to mutations *in vivo*. Prenatal exposure to 1 Gy of acute irradiation on GD12 resulted in a 1.94-fold increase in the ESTR mutation rate [22].

NanoTiO_2_ can induce DNA strand breaks and carcinogenic effects *in vivo*[11,25,26]. We recently reported that UV-Titan inhalation did not increase DNA strand breaks in the P or F1 generations [10], suggesting that genotoxic effects in offspring are negligible. Correspondingly, in the study of prenatal DEP exposure by [2], which showed ESTR instability in male offspring, the exposure also failed to increase DNA strand breaks in liver from newborns [27]. Epigenetic changes have been suggested as the underlying mechanism of ESTR instability [5,22]. A recent study found DNA deletions in mice prenatally exposed to nanoTiO_2_[26]. However, the small effective sample size and the very large maternal dose used in the study hamper interpretation. The results on nanoTiO_2_ induced mutations and genotoxicity are conflicting [10,15,26]. The various types of commercially available nanoTiO_2_ also make it difficult to generalize. It is possible that NPs with very active surface chemistry, which produce more reactive oxygen species (ROS) or a large inflammatory response, could induce germline mutations. In the present study we have only assessed the effects of a single type of TiO_2_ NP. We are currently investigating the effects of prenatal exposure to nanosized carbon black Printex90, a more efficient generator of ROS than both DEP and nanoTiO_2_[12] to further address the question of female susceptibility to NPs. The present study indicates that prenatal exposure to nanoTiO_2_ does not affect female germline ESTR mutation frequency.

## Competing interests

The authors declare that they have no competing interests.

## Authors’ contributions

AMZB was substantially involved in the design of the study, collected animal tissue, processed samples and performed the electrophoresis, blot probing, image processing, mutation scoring, statistical analysis and drafted the manuscript. TS re-probed and developed images for a large portion of blots and revised the manuscript. PJ exposed the P generation mice, assigned F1 offspring for the current study and revised the manuscript critically. KSH was project manager of the study and revised the manuscript critically. HW was substantially involved in the design of the study and revised the manuscript critically. CLY was substantially involved in the design of the study, scored mutations and revised the manuscript critically. UBV was substantially involved in the design of the study and revised the manuscript critically. All authors read and approved the final version of the manuscript.
